# Overexpression of *Mtr-miR319a* Contributes to Leaf Curl and Salt Stress Adaptation in *Arabidopsis thaliana* and *Medicago truncatula*

**DOI:** 10.3390/ijms24010429

**Published:** 2022-12-27

**Authors:** Mingna Li, Lei Xu, Lixia Zhang, Xiao Li, Chunyu Cao, Lin Chen, Junmei Kang, Qingchuan Yang, Yajiao Liu, Bilig Sod, Ruicai Long

**Affiliations:** Institute of Animal Sciences, Chinese Academy of Agricultural Sciences, Beijing 100193, China

**Keywords:** *Medicago truncatula*, *miR319*, salt tolerance, TCP, leaf curl

## Abstract

Salt stress is a worldwide agronomic issue that limits crop yield and quality. Improving salt stress tolerance via genetic modification is the most efficient method to conquer soil salinization problems in crops. Crop miRNAs have been declared to be tightly associated with responding and adapting to salt stress and are advantageous for salt tolerance modification. However, very few studies have validated vital salt tolerance miRNAs and coupled potent target genes in *Medicago* species, the most economically important forage legume species. In this study, *Mtr-miR319a*, a miRNA that was identified from the previous next-generation sequencing assay of salt-treated *Medicago truncatula*, was overexpressed in *M. truncatula* and *Arabidopsis thaliana*, inducing the curly leaves and salt stress tolerance phenotypes. Combining the elevated expression level of *Mtr-miR319a* in the *M. truncatula* overexpression lines under normal and salt-treatment conditions, the regulatory roles of *Mtr-miR319a* in leaf development and salt stress adaptation were demonstrated. Several predicted target genes of *Mtr-miR319a* were also regulated by *Mtr-miR319a* and were associated with the aforementioned phenotypes in *M. truncatula* plants, most notably *MtTCP4*. Our study clarified the functional role of *Mtr-miR319a* and its target genes in regulating leaf development and defending salt stress, which can help to inform crop breeding efforts for improving salt tolerance via genetic engineering.

## 1. Introduction

Soil salinization is a worldwide issue affecting crop yield, quality, and distribution [1,2]. When plants are exposed to a salt circumstance, various biological processes are negatively impacted by osmotic stress, ion toxicity, and oxidative damage due to the salinity [3,4]. It is calculated that more than half of the arable land in the world will suffer from salinity in 30 years [5]. Hence, studying salt stress adaptation mechanisms and enhancing the salt tolerance of plants are important and imperative for crop productivity and agricultural sustainable development around the world.

MicroRNAs (miRNAs), which are widely distributed in diverse plant species, are endogenous non-coding RNA transcripts with a small length of 19–24 nt [6,7]. miRNAs have a vital role in the regulation of gene transcription, cell proliferation, and protein translation [6,8]. Under salt stress, miRNAs function by activating rapid and synchronized changes at post-transcriptional levels for responding to the detrimental circumstance [9,10]. Currently, there are significant efforts to identify stress-inducible miRNAs and miRNA target genes via high-throughput sequencing approaches [6,9,11,12]. For instance, several salt stress-related miRNAs, which could have a broad range of target mRNAs and perform important roles in salt stress regulation, were documented in alfalfa (*M. sativa*) and *M. truncatula* [6,9], whereas the overexpression of *miR393* can enhance salt stress adaptation and ABA insensitivity in *A. thaliana* [10]. Accordingly, understanding and modifying the expression level of critical miRNAs coupled with their potential targets is an efficient way to improve the salinity tolerance of important crops [12].

*MicroRNA319* (*miR319*) is one of the most original and conserved miRNA families and is a crucial regulator of leaf development and growth [13,14]. Enhanced expression levels of *miR319* can downregulate *teosinte-branched/Cycloidea/proliferating (TCP) cell factor* genes, which leads to changes in leaf morphogenesis in *A. thaliana* [13,15]. In Chinese cabbage (Brassica rapa), overexpressing *BrpMIR319a2* decreases the expression of *BrpTCP4*, leading to the excessive expansion of both the topmost and interveinal regions and the enlargement of cylindrical cabbage heads [16,17]. By inhibiting the function of targeted TCP genes, the overexpression of *miR319* also positively regulates the expression of *cup-shaped cotyledon* (*CUC*) genes, which are also active in the sinuses of the leaf margin and are required for leaf serration [18,19]. Furthermore, *miR319* was also found to be enhanced in *A. thaliana* [20], wheat (*Triticum aestivum* L.) [21], and switchgrass (*Panicum virgatum* L.) [22] and downregulated in maize (*Zea mays* L.) [23] and *Solanum linnaeanum* [12] under high salt stress conditions, indicating a diverse regulatory role for *miR319* in different developmental stages, tissues, and species during salt stress responses in plants. Moreover, the myeloblastosis (MYB) transcription genes in switchgrass (*Panicum virgatum* L.) [24] and watermelon (*Citrullus lanatus*) [25], TCP transcription factor genes in creeping bentgrass (*Agrostis stolonifera*) [26], and the lipoxygenase (LOX) gene [27], which *miR319* targets, are reported to regulate salt tolerance. Therefore, *miR319* is an essential candidate for genetic modification for the improvement of the growth and development of crops grown under high salt conditions.

*Medicago* species, the most important legume crop species around the world, play a significant agronomic and ecologic role due to the fact of its high protein and nutritional value and symbiotic nitrogen fixation [28]. Being a universal *Medicago* model plant with salt-sensitive properties, *M. truncatula* can be advantageous for the molecular investigation of the salt stress mechanism and salt tolerance modification of *Medicago* species. Notably, in our previous studies on *M. truncatula*, *miR319* was demonstrated to participate in responding to salt stress, using high-throughput deep-sequencing approaches [6,9], indicating the possible role that *miR319* could have in the *Medicago* species. However, knowledge on the further administrative roles of *miR319* in adapting to salinity is very inadequate in *Medicago* species.

The hypothesis of this work was that *miR319* could contribute to salt stress adaptation and tolerance via targeting several potential genes such as *TCP4* in *Medicago* species. Accordingly, in this study, *Mtr-miR319a* (*miR319a* from *M. truncatula*) was overexpressed in *A. thaliana* and *M. truncatula* plants, which was followed by assays on the growth and physiology for metrics evaluation, targets prediction, and expression detection of *Mtr-miR319a* and its targets under control and salt-treated conditions. The objective was to analyze the specific role of *Mtr-miR319a* and its potential targets during development and salt stress and to test whether heightening the expression of *Mtr-miR319a* can modify salt tolerance abilities in *M. truncatula*. The study manifested the leaf margin formation and salt tolerance function of *Mtr-miR319a* in *M. truncatula* and verified its crucial target gene, *MtTCP4*, which would help to promote the genetic engineering and modification of the salt tolerance ability in *Medicago* and other plant species.

## 2. Results

### 2.1. Sequence Analysis of Mtr-miR319a

**Figure 1:**
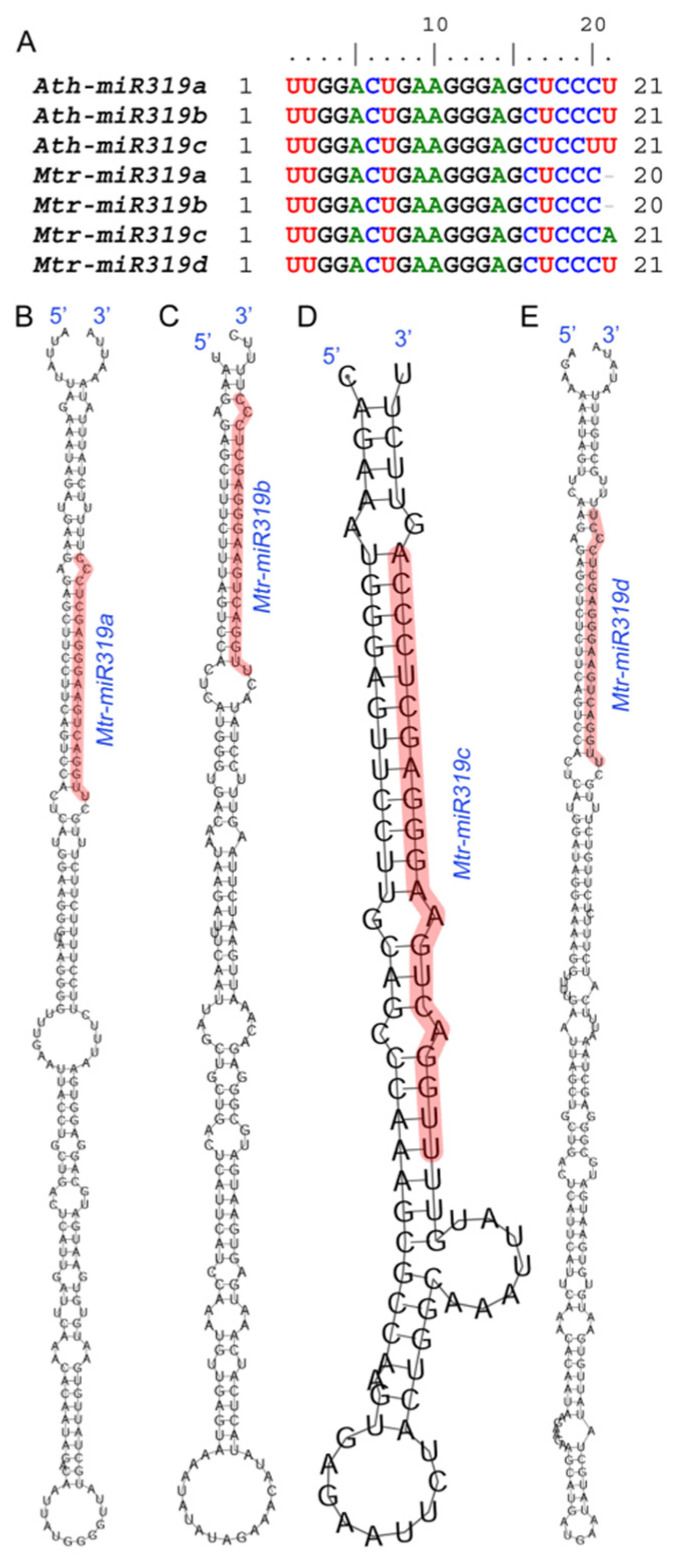
Sequence analysis of *miR319* and precursor secondary structure prediction: multiple sequence alignment of *miR319* in *A. thaliana* and *M. truncatula* (**A**), *A. thaliana* sequences of *Ath-miR319a*, *Ath-miR319b* and *Ath-miR319c*, and *M. truncatula* sequences of *Mtr-miR319a*, *Mtr-miR319b*, *Mtr-miR319c* and *Mtr-miR319d* were aligned; secondary structure prediction of the precursors of *Mtr-miR319a* (**B**), *Mtr-miR319b* (**C**), *Mtr-miR319c* (**D**), and *Mtr-miR319d* (**E**). The mature sequences of Mtr-miR319s are marked in red.

To explore the sequence conservation and variation of *miR319* in *A. thaliana* and *M. truncatula*, multiple sequence alignment of the mature sequences and the secondary structure prediction of the precursors were analyzed. The results of the multiple sequence alignment showed that the mature sequences of *miR319* in *A. thaliana* and *M. truncatula* were conserved (Figure 1A), indicating that the overexpression of *Mtr-miR319a* in *A. thaliana* might work normally. However, the results also showed that there were differences among the sequences as well as the secondary structures of *Mtr-miR319* precursors in *M. truncatula* (Figure 1B).

### 2.2. Leaf Curl Phenotypes in Mtr-miR319a-Overexpressed A. thaliana and M. truncatula Plants

**Figure 2:**
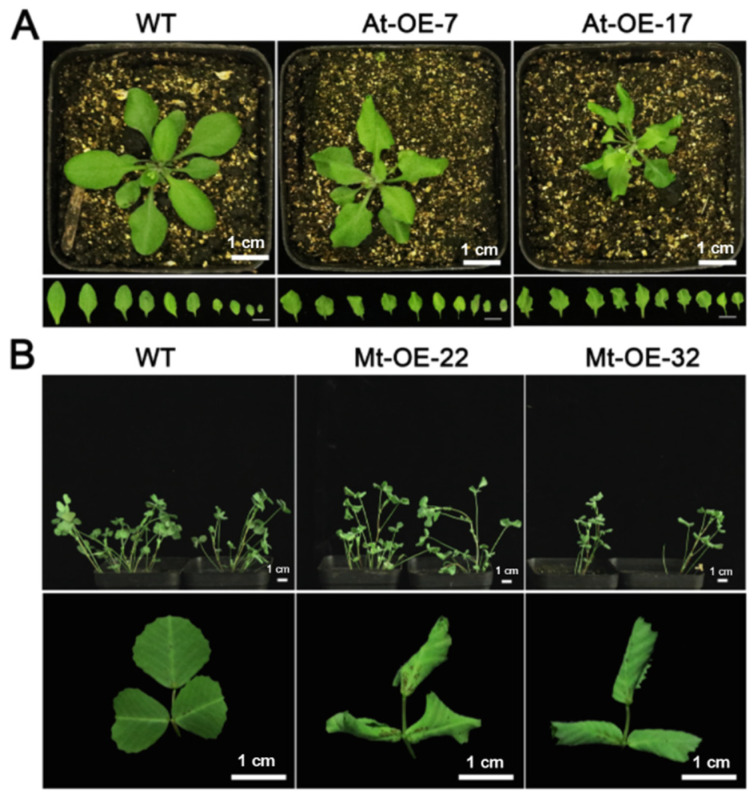
Phenotypes of leaf curl after overexpressing *Mtr-miR319a*: phenotypes in the leaves of *A. thaliana* (**A**) and *M. truncatula* (**B**) of the WT and *Mtr-miR319a*-overexpressing plants. Mature WT *A. thaliana* plants and *Mtr-miR319a*-overexpressing plants of At-OE-7 and At-OE-17, as well as mature WT *M. truncatula* plants and *Mtr-miR319a*-overexpressing plants of Mt-OE-22 and Mt-OE-32 are shown. Bar = 1 cm.

The *Mtr-miR319a*-overexpressed *A. thaliana* lines of At-OE-7 and At-OE-17 and *M. truncatula* lines of Mt-OE-22 and Mt-OE-32 were used for further functional clarification (Figure 2). In the *Mtr-miR319a*-overexpressed *A. thaliana* seedlings, both young and mature leaves were curled with an impaired cotyledon boundary and serration formation, which could also be found in the *Mtr-miR319a*-overexpressed *M. truncatula* seedlings (Figure 2). Except for this leaf curl phenotype, no other growth or developmental differences were found in comparison to the WT plants in both *A. thaliana* and *M. truncatula*. The leaf development performance in the overexpression plants might be caused by the overexpression of *Mtr-miR319a*.

### 2.3. Overexpression of Mtr-miR319a and Its Impacts on Salt Tolerance in A. thaliana and M. truncatula

**Figure 3:**
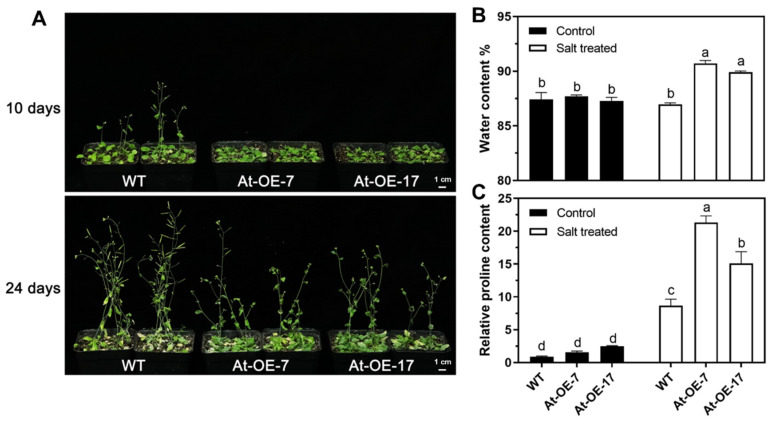
Growth and physiological differences of *Mtr-miR319a* overexpressing *Arabidopsis* plants after salt stress: Phenotype (**A**), physiology indicators of water content (**B**), and relative proline content (**C**) of *Arabidopsis* plants overexpressing *Mtr-miR319a* after salt stress. The WT *Arabidopsis* plants, *Mtr-miR319a*-overexpressing plants of At-OE-7 and At-OE-17 were exposed to salt stress of 200 mM NaCl for 10 d (**A**) and 24 d (**A**–**C**). The values are shown as mean ± standard error (SE); n = 3 for all groups. The bars represent the SE. Bars with different lowercase letters indicate statistically significant differences at *p* < 0.05 based on ANOVA. Bar = 1 cm.

**Figure 4:**
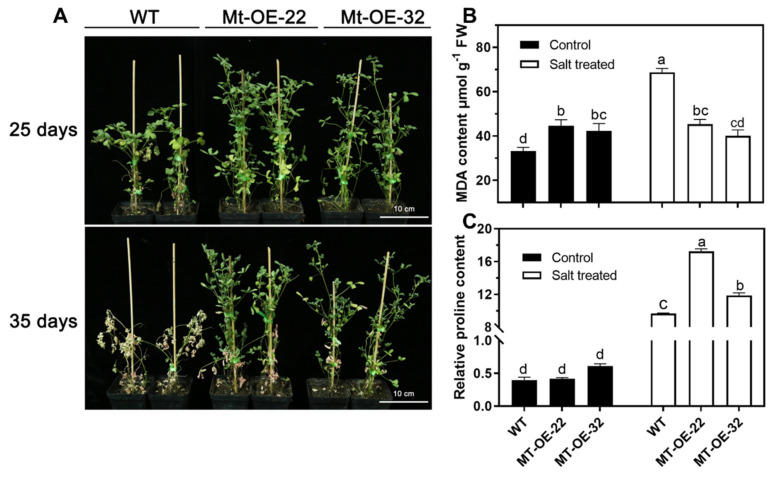
Growth and physiological differences of *Mtr-miR319a*-overexpressed *M. truncatula* plants after salt stress: phenotype (**A**); physiological indicators of MDA content (**B**); relative proline content (C) of *M. truncatula* plants overexpressing *Mtr-miR319a* after salt stress. The WT *M. truncatula* plants and *Mtr*-*miR319a*-overexpressing lines of Mtr-OE-22 and Mtr-OE-32 were exposed to salt stress of 200 mM NaCl for 25 d (**A**) and 35 d (**A**–**C**). The values are shown as the mean ± standard error (SE); *n* = 3 for the groups. The bars represent the SE. Bars with different lowercase letters indicate statistically significant differences at *p* < 0.05 based on ANOVA. Bar = 10 cm.

To test the salt tolerance ability of the *Mtr-miR319a*-overexpressing plants, growth and physiological assays were conducted in *A. thaliana* and *M. truncatula* plants under salt stress (Figure 3 and Figure 4). After exposure to high salt conditions for 24 and 35 d in *A. thaliana* and *M. truncatula*, respectively, the growth status of all of the overexpressed plants was better than their corresponding WT plants (Figure 3A and Figure 4A). The original plant status before salt treatment at 0 d is shown in Figure 2. Specifically, in *A. thaliana*, the water content of At-OE-7 and At-OE-17 was significantly higher than that of the WT plants under salt stress, which showed no difference under the control conditions (Figure 3B). The relative proline content was higher in the At-OE-7 and At-OE-17 plants compared to the WT plants under salinity (Figure 3C). In *M. truncatula*, the MDA content in the overexpression lines did not change after salt stress, whereas it was significantly higher in the WT plant (Figure 4B), and the relative proline content was significantly increased after salt stress while the two overexpression plants had higher levels than the WT plants (Figure 4C). The growth and physiological performances of the WT and *Mtr-miR319a* overexpression plants after salt stress demonstrated that overexpressing *Mtr-miR319a* is beneficial for improving salt tolerance in *A. thaliana* and *M. truncatula*.

### 2.4. High Expression of Mtr-miR319a Was Maintained before and after Salt Stress in Mtr-miR319a-Overexpressed M. truncatula Plants

**Figure 5:**
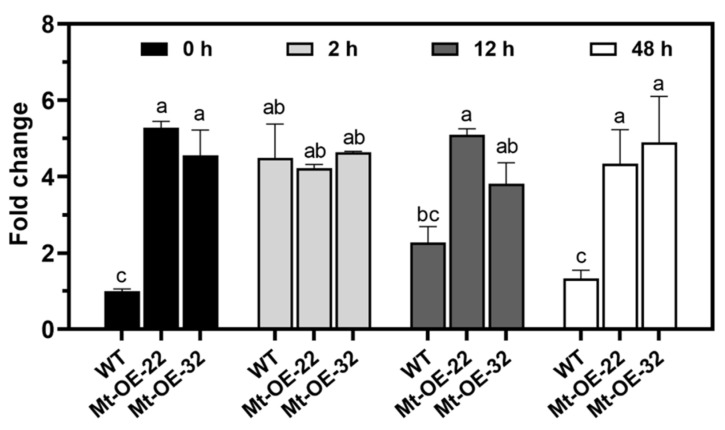
Transcriptional expression profile of *Mtr-miR319a* in *M. truncatula* plants after salt stress. The *M. truncatula* plants of the WT and the *Mtr-miR319a* overexpression lines of Mtr-OE-22 and Mtr-OE-32 were exposed to 150 mM NaCl for 0, 2, 12, and 48 h. The values are shown as the mean ± standard error (SE); *n* = 3 for all groups. The bars represent the SE. Bars with different lowercase letters indicate statistically significant differences at *p* < 0.05 based on ANOVA.

To explore the expression pattern of *Mtr-miR319a* after salt circumstance, the expression level of *Mtr-miR319a* in *M. truncatula* WT and *Mtr-miR319a*-overexpressing plants was determined after exposure to a time course of salt treatment (Figure 5). The results showed that the expressions of *Mtr-miR319a* in Mt-OE-22 and Mt-OE-32 were significantly higher than that of the WT seedlings at 0 h, showing five-fold differences. The expression of *Mtr-miR319a* in the WT plants increased five-fold after 2 h of salt treatment, which showed no difference compared to the overexpression plants. However, the expression of *Mtr-miR319a* in the WT plants increased two-fold after 12 h of salt treatment and did not show significant changes at 48 h compared to its value at 0 h, whereas the overexpression plants did not significantly change during the 48 h. The rapid induction of *Mtr-miR319a* in the WT indicates that the salt-responsive role of *Mtr-miR319a* in *M. truncatula* and the constantly high expression (compared to the WT plants in 0 h) over the 48 h could contribute to salt tolerance in the *Mtr-miR319a M. truncatula* overexpression plants.

### 2.5. Mtr-miR319 Acts on the Target Genes Involved in Leaf Development and Salt-Responsive Roles in M. truncatula

**Figure 6:**
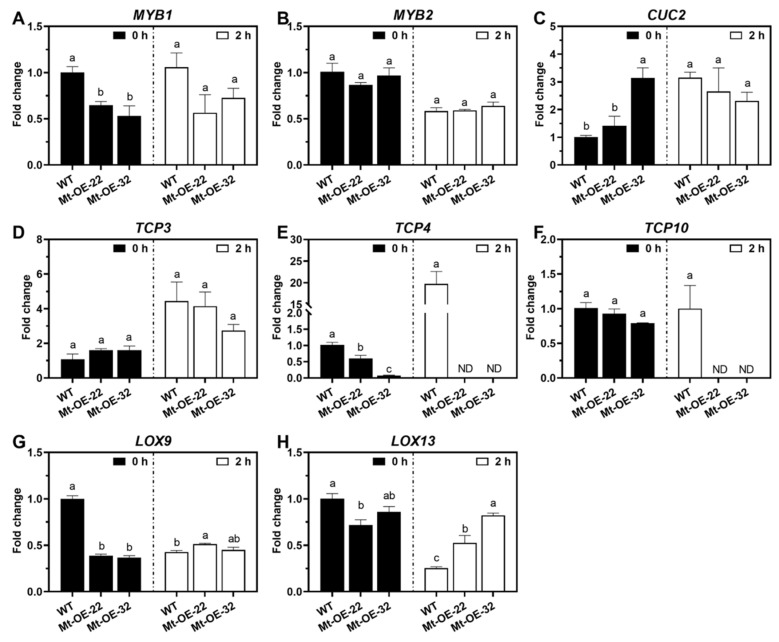
Transcriptional expression analysis of the predicted target genes of *Mtr-miR319a* in *M. truncatula* plants before and after salt stress. The predicted target genes of *MYB1* (**A**), *MYB2* (**B**), *CUC2* (**C**), *TCP3* (**D**), *TCP4* (**E**), *TCP10* (**F**), *LOX9* (**G**), and *LOX12* (**H**) were detected. Twenty-eight-day-old WT *M. truncatula* plants and *Mtr-miR319a*-overexpressing lines of Mtr-OE-22 and Mtr-OE-32 were detected after exposure to 150 mM NaCl for 0 and 2 h. “ND” represents “Not detected”. The values are shown as the mean ± standard error (SE); n = 3 for all groups. The bars represent the SE. Bars with different lowercase letters indicate statistically significant differences at *p* < 0.05 based on ANOVA.

To examine the transcriptional expression of the possible targets for verifying the functional roles of *Mtr-miR319*, eleven genes, including *MYBs*, *CUC*, *TCPs*, and *LOXs*, which are involved in leaf development and salt stress adaptation, were determined before and after salt stress in *M. truncatula* plants (Figure 6). Specifically, the expression of *MYB2* and *TCP3* did not show a significant difference between the WT and the overexpression lines under both control and salinity circumstances. Under normal conditions, the expression of *LOX13* in Mt-OE-22 and *MYB1*, *TCP4*, and *LOX9* in the overexpression lines were significantly suppressed by *Mtr-miR319*, while *TCP10* in the overexpression lines and *LOX13* in Mt-OE-32 did not change. In addition, the *CUC2* levels in Mt-OE-32 significantly increased. However, after 2 h of salt treatment, the expression of *TCP4* and *TCP10* in the overexpression lines sharply declined, *MYB1* and *CUC2* in the two overexpression lines did not show any difference with the WT, and *LOX9* in Mt-OE-22 and *LOX13* in the two overexpression lines were significantly higher than in the WT plants. According to the expression changes, it can be found that *MYB1*, *TCP4*, *TCP10*, and *LOX9* were suppressed under normal or salt stress conditions, indicating the inhibitory role that *Mtr-miR319a* plays on these target genes.

### 2.6. Prediction and Verification of the Target Genes of Mtr-miR319a

**Figure 7:**
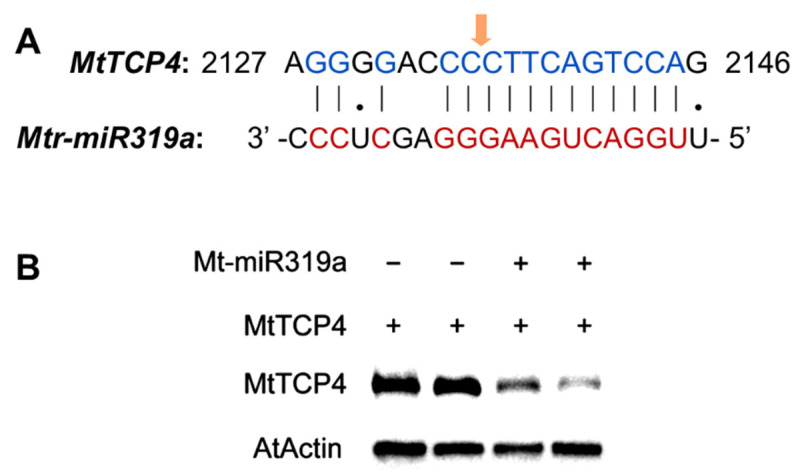
Prediction and verification of the cleavage of *MtTCP4*, the target gene of *Mtr-miR319a*: prediction of the cleavage of *MtTCP4* as the target gene of *Mtr-miR319a* (**A**); verification of the cleavage of MtTCP4, the target gene of *Mtr-miR319a*, by Western blot (**B**). The orange arrow in (**A**) indicates the site where the target gene was cleaved by *Mtr-miR319a*.

*MtTCP* was found to be a primary target gene of miR319 by using the psRNATarget miRNA target gene analysis server (http://plantgrn.noble.org/psRNATarget/home, accessed on 22 January 2021) [29]. Specifically, *MtTCP4* (*MTR_8g463380*) was strongly conserved with the predicted binding region, whereas the combination of *Mtr-miR319a* and *MtTCP4* (*MTR_8g463380*) completely matched. The results of 5′ RLM-RACE showed that the cleavage sites occurred at the 9th to 10th base sites of *Mtr-miR319a* in *MtTCP4* (Figure 7A). Furthermore, the Western blot assay showed that the protein translation of MtTCP4 was suppressed by *Mtr-miR319a* (Figure 7B). Thus, this work predicted and verified the target gene *MtTCP4* of *Mtr-miR319a*.

## 3. Discussion

**Figure 8:**
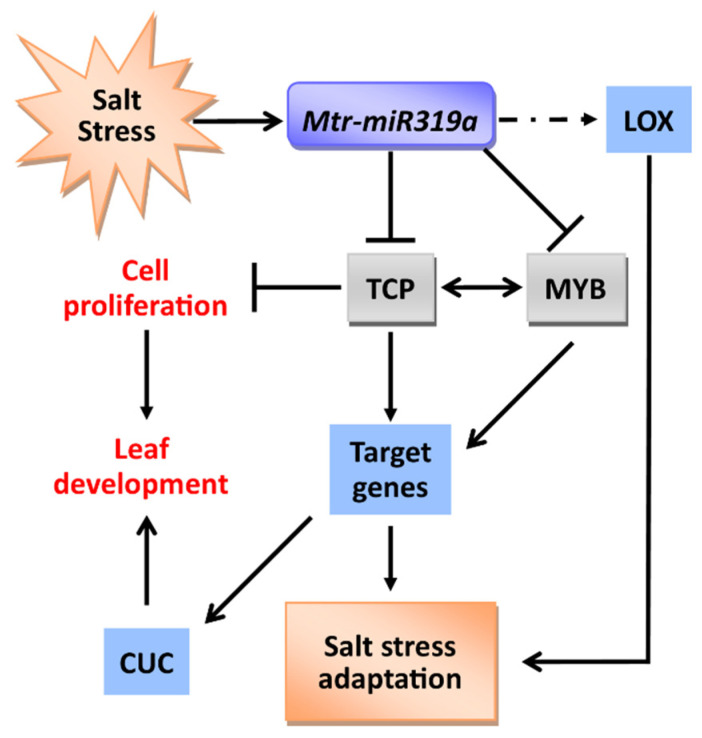
Hypothetical model showing the molecular regulatory mechanism of *Mtr-miR319a* in defending salt stress and affecting leaf development. Lysyl oxidase (LOX); teosinte-branched/Cycloidea/proliferating (TCP); cup-shaped cotyledon (CUC); myeloblastosis (MYB).

Salinity is a widespread problem that threatens the yield and production of crops across the world [30]. Exploring salt response and tolerance mechanisms can lay the theoretical foundation for salt tolerance genetic improvement and modification of economically important crops. MicroRNAs have been shown to play an essential role in the regulation of gene transcription, protein translation, and cell proliferation [6,8]. Herein, our study firstly verified that the overexpression of *Mtr-miR319a* can regulate leaf development and salt tolerance via targeting several critical genes (Figure 8), such as *TCP*, *MYB*, *CUC*, and *LOX*, in *M. truncatula* plants, which is discussed below.

In plants, leaves are the most essential plant organs for energy acquisition and carbohydrate generation. Crinkled and curly phenotypes were found in the leaves of the *Mtr-miR319a*-overexpressing *A. thaliana* and *M. truncatula* plants in our results. This conspicuous leaf morphology was also reported in *miR319*-overexpressing petunia (*Petunia* × *hybrida*) [19] and tomato (*Solanum lycopersicum*) [31] and in *tcp A. thaliana* mutants [32]. In our study, the verified *Mtr-miR319a* target gene, *TCP4*, was detected to be suppressed under normal conditions in the *Mtr-miR319a*-overexpressed *M. truncatula* plants. This is in accordance with the observation that overexpressing *miR319* can reduce the expression levels of *TCPs* and trigger leaf serration in *A. thaliana*, whereas the *mir319a/b* mutant enhanced the expression of *TCPs*, resulting in the formation of smooth leaves [15,18]. Therefore, our results suggest that the robust and intricate roles of *Mtr-miR319a* and its targets, namely, TCP transcription factors, play a crucial role in the development process of the central and marginal regions of the leaf of *A. thaliana* and *M. truncatula* [19,32]. In addition, TCP4 negatively regulates the expression of *CUC* genes and directly interacts with CUC2, which is also involved in leaf serration formation [18,33]. The expression of *CUC2* was significantly higher in one of the overexpression lines. This further indicates that the regulatory networks of *Mtr-miR319a* and its target genes act on leaf formation and development [33].

Notably, the leaf phenotype caused by *Mtr-miR319a* can contribute to improving salt tolerance. Wider, thicker leaves with increased weight-to-area ratios have been reported in *miR319*-overexpressing creeping bentgrass plants [26] and switchgrass [24], and they were tightly associated with enhanced salt tolerance by means of maintaining higher water contents, photosynthetic activity, and stomatal conductance, reducing ROS levels (represented by H_2_O_2_ content) and accumulating less Na^+^ when coping with salt stress [24,26].

Though the *miR319* network in plants is evolutionarily conserved, *miR319*-targeted genes might have conflicting functions between dicotyledonous and monocotyledonous plant species [34]. Therefore, this could explain why the leaf phenotypes in the *A. thaliana* and *M. truncatula* plants in this study were not identical to those in creeping bentgrass and switchgrass, which are monocotyledons in the *Poaceae* family and are inherently anatomically different from the plant materials used in the present study [34]. Despite these morphological differences, the physiological and biochemical parameters also exhibited improved salt tolerance in the *Mtr-miR319a*-overexpressing *A. thaliana* and *M. truncatula* plants in our study, indicating that the regulatory role of *Mtr-miR319a* could also be associated with salt tolerance but is not limited to impacting leaf development and morphology [34].

We noticed a delayed flowering time and the suppression of leaf senescence in the *Mtr-miR319a*-overexpressed *A. thaliana* and *M. truncatula* plants when exposed to salt stress. This phenotype was also reported in *miR319*-overexpressed creeping bentgrass [26] and tomato plants [31]. In this study, the delayed flowering and leaf senescence phenotypes under salt stress could indicate a role of *Mtr-miR319a* in flowering time and leaf senescence regulation [35], which also contribute to the salt tolerance performance caused by *Mtr-miR319a*, as manifested in *A. thaliana* [36,37].

Furthermore, the expression of *LOX9* was found to be repressed in *Mtr-miR319a*-overexpressing *M. truncatula* plants under normal conditions, whereas the expression of *LOX9* in Mt-OE-22 and *LOX13* in the two overexpression lines were identified to be significantly higher under salt stress compared to the WT plants. *LOX*, which encodes the key enzymes of JA biosynthesis and catalyzes the reaction of α-linoleic acid to hydroperoxy-octadecadienoic acid, was induced in *MIR319b* overexpression rice plants upon blast disease exposure [38]. For the *M. truncatula* plants coping with salt stress in our study, the expression levels of *LOX9* in Mt-OE-22 and *LOX13* in the overexpression plants were also higher than that of the WT plant. This induction could be explained by the role of *Mtr-miR319a* targeting genes used in salt stress defense in *M. truncatula* plants [38]. In addition, a previous study [39] demonstrated that TCP4 adjusts leaf senescence via binding to the *LOX2* promoter and commanding the transcriptional level of *LOX2*. In this study, the roles of improving salt tolerance and regulating leaf senescence by interacting with TCP4 in *LOX9* and *LOX13* warrant further clarification.

Moreover, *GaMYBs*, gibberellin and abscisic acid-regulated *MYBs*, are reported to be targeted by *miR319* and can improve chilling [40] and heat [41] stress tolerance in tomato. The expression of the predicted *Mtr-miR319a* target gene, *MYB1*, was found to be suppressed in the *Mtr-miR319a*-overexpressed *M. truncatula* plant (Mt-OE-32) under normal conditions compared to the WT plants. The expression of *OsGAmyb* was significantly lower in the *miR319a* overexpression plants [42], which is in accordance with our determined expression changes of *MYB1*, indicating that *MYB1* could also be a target gene of *Mtr-miR319a* in the defense against salt stress in *M. truncatula* plants.

## 4. Materials and Methods

### 4.1. Plant Materials and Culture Environments

*A. thaliana* (Col-0) and *M. truncatula* (R108) were taken as wild-type (WT) plants in the following experiments. *A. thaliana* and *M. truncatula* seedlings were cultured under identical environmental conditions in artificial climate incubators (GXZ-500, Jiangnan, China) with the environmental conditions of 20 °C, 16/8 h light/dark, 65% humidity, and 125 μmol m^−2^ s^−1^. The transgenic seeds of *A. thaliana* obtained from individual plants were disinfected by immersion in 5% (volume) NaClO solution, rinsed with sterilized distilled water, spread onto half Murashige and Skoog (MS) medium in plates (d = 10 cm), and reserved at 4 °C for two days. The transgenic seeds of *M. truncatula* obtained from individual plants were sterilized as described for the *A. thaliana* seeds and placed in plates (d = 10 cm) with a filter paper for seed germination. For the seedling growth assays under salt stress, the 10 day transgenic *A. thaliana* and *M. truncatula* seedlings were transferred to pots (9 × 9 × 12 cm) filled with soil (2:1 mixture of nutrient soil and vermiculite). For the transcriptional expression analysis, the 2 week-old *M. truncatula* seedlings were replanted in hydroponic culture containers (25 × 20 × 7.5 cm) in 2.2 L of half-strength Hoagland nutrient solution in the growth chamber for 28 d. The half-strength Hoagland nutrient solution was replaced every 4 d to keep fresh.

### 4.2. Salt Treatments and Sampling

For seedling growth under salt stress tests, four-week-old WT and *Mtr-miR319a*-overexpressing *A. thaliana* seedlings were irrigated with 20 mL of 200 mM NaCl solution every 3 d for 24 d, and the leaves were sampled at 24 d for the physiological measurements; the four-week-old WT and *Mtr-miR319a*-overexpressed *M. truncatula* plants were exposed to 20 mL of 200 mM NaCl solution every 3 d for 35 d, and the leaves were sampled at 35 d for the physiological measurements. For the transcriptional analysis, the hydroponic solution was supplemented with 150 mM NaCl. Twenty-eight-day-old *M. truncatula* plants were treated with 150 mM NaCl, and leaf samples were collected at 0, 2, 12, and 24 h, respectively; rinsed with deionized water; frozen in liquid nitrogen immediately; and kept at −80 °C until nucleic acid isolation. Each time point was duplicated three times, and each replicate pooled three individual plants.

### 4.3. RNA/DNA Isolation, cDNA Synthesis, and 5′ RLM-RACE

The total RNA was isolated from the *M. truncatula* leaves using Trizol solution (Invitrogen, Carlsbad, CA, USA). The total RNA used for the reverse transcription was first treated with DNase I enzyme (MBI Fermentas, Hanover, MD, USA). The PrimeScript Reverse Transcriptase Kit (Takara, Japan) was used for the cDNA synthesis reaction, referring to the manufacturer’s instructions. The genomic DNA extraction was carried out using a DNA isolation kit (Kangweishiji, Beijing, China) according to the manufacturer’s instructions. The harvested complementary cDNA and DNA samples were well stored at −20 °C in a freezer. The cleavage sites of the miRNA targets in *M. truncatula* were conducted with 5′ RNA ligase-mediated rapid amplification of the cDNA ends (5′ RLM-RACE) [43] using the SMARTer^®^ RACE 5′/3′ Kit (Takara, Kyoto, Japan). The synthesized cDNA was taken as a template for the PCR with 5′ primer and gene-specific primers. The gene-specific primers (GSPs) were validated at the 5′ end of the RNA adaptor, and the amplified universal primers complementary to the adaptor were designed, as listed in Table S1. The procedures for the RNA adaptation, reverse transcription, and amplification followed the recommended instructions (Kangweishiji, Beijing, China). The product was purified and transformed into the EASY-T5 vector (TransGen, Beijing, China) for sequencing (Zhongchuanhongda, Beijing, China) and was preserved for further usage after the sequence alignment and verification.

### 4.4. Overexpression Vector Construction and Genetic Transformation

The precursor sequences of *Mtr-miR319a* were searched using the miRbase database (http://www.mirbase.org/, accessed on 22 January 2021). The cloning primers, Mtr-miR319a-F/R, were designed using Pimer6.0 software according to the sequences of the plasmid and precursor, as displayed in Table S1. The amplification products were detected on 1% agar gel stained with gold view (Transgen, Beijing, China) and then purified with a DNA gel extraction kit (Transgen, Beijing, China). The purified fragment was concatenated into the pCAMBIA3301 plasmid that was previously digested with the *Nco*I restriction enzyme. The pCAMBIA3301-*Mtr-miR319a* recombinant plasmid was preserved after sequencing (Qingkexinye, Beijing, China). *MtTCP4* was cloned by GXL DNA Polymerase (TaKaRa, Kusatsu, Shiga, Japan), ligated into the PCR-TOPO vector (Invitrogen, Waltham, MA, USA), and converted into competent *E. coli*. After sequencing and vector extraction, TOPO-*MtTCP4* was obtained. The recombination of TOPO-*MtTCP4* and the pMDC83 vector was performed by Gateway^®^ LR Clonase^®^ II (Invitrogen, Waltham, MA, USA) following the operational steps. The pMDC83-*TCP4* recombinant plasmid was finally obtained and well preserved after sequencing (Qingkexinye, Beijing, China).

The pCAMBIA3301-*Mtr-miR319a* plasmid was transformed into *A. thaliana* following the *Agrobacterium* (GV3101)-mediated floral-dipping method [44]. The harvest-transformed *A. thaliana* seeds were further spread on 1/2 MS medium with glufosinate (PPT) (4 mg/L) application and verified by PCR and qRT-PCR assays for the positive lines. The pCAMBIA3301-*Mtr-miR319a* vector was transformed into *M. truncatula* using a unified *Agrobacterium*-mediated genetic transformation protocol [45]. The callus was induced and cultured on SH3a medium with PPT (3 mg/L) and cephalosporin (CEP) (450 mg/L) for de-the differentiation, MSBK with PPT (3 mg/L) and CEP (450 mg/L) for the embryo induction, and SH9a with PPT (1 mg/L) and CEP (200 mg/L) for the shoot and root induction. The generated *M. truncatula* seedlings were also positively selected using PCR and qRT-PCR assays. The pMDC83-*TCP4* vector was transiently transformed into the tobacco leaves mediated by *Agrobacterium* GV3101 [46], and the proteins of the GFP fusion and control were examined by applying a confocal scanning microscope system (Leica TCS SP8, Germany).

### 4.5. Physiological Measurements for Salt Tolerance Evaluation

The physiological parameters, such as relative water content [47], malondialdehyde (MDA) content [48], and relative proline content [49], of *A. thaliana* and *M. truncatula* plants after the treatments were determined. The samples for the relative water content and relative proline content measurements were taken from 3 independent biological duplicates for each sampling, while four were used for the MDA content.

### 4.6. Western Blot Analysis for the Mtr-miR319a Target Protein

The total protein was separated from the transformed tobacco leaves through the adoption of the isolation buffer, and the protein concentration was measured using the blood alcohol concentration method. The protein samples were boiled for 12 min after mixing with the buffer. The lysates were split by SDS-PAGE and checked by immunoblotting against the rabbit anti-GFP antibody (Abcam, ab290, Cambridge, MA, USA) for GFP-MtTCP4. As a loading control, actin was detected with the rabbit anti-Actin antibody (Abcam, ab197345, Cambridge, MA, USA). The HRP-conjugated goat anti-rabbit secondary antibody (Beyotime, A0216, Beijing, China) was adopted for anti-GFP or anti-actin immunoblotting.

### 4.7. RT-qPCR Assay of Mtr-miR319a and Its Targets

Relative quantification analyses of *Mtr-miR319a* and its target genes of *MYB1 (MTR_3g011610)*, *MYB2 (MTR_8g042410)*, *CUC2 (MTR_2g078700)*, *TCP3 (MTR_2g078200)*, *TCP4 (MTR_8g463380)*, *TCP10 (MTR_2g090960)*, *LOX9 (MTR_8g018690)*, and *LOX13 (MTR_3g479460)* were conducted on a real-time fluorescent quantitative ABI 7300 PCR system (Applied Biosystems, Foster City, CA, USA) and estimated referring to the comparative Ct method. The *MtU6 snRNA* and *MtActin2* genes were taken as the house-keeping reference genes of *Mtr-miR319a* and its target genes, respectively. The qRT-PCR primers were prepared at the NCBI (http://www.ncbi.nlm.nih.gov/tools/primer-blast/, accessed on 15 June 2021) (Table S1). Reactions were conducted using the miCute enhanced miRNA fluorescence quantitative assay kit (Tiangen, Beijing, China) and SYBR Premix Ex TaqTM II (TaKaRa, Kusatsu, Shiga, Japan), and the quantitative tests were replicated three times. The PCR cycling conditions were set on the basis of the manufacturer’s guidance, with a melting curve to affirm the product specificity and avert the primer dimers in the end. The relative expressions of Mtr-miR319a and each gene were analyzed referring to the 2^−∆∆Ct^ formula [50] and presented as the fold change.

### 4.8. Statistics

The data analysis was conducted using EXCEL 2010 (Microsoft Corporation, Redmond, WA, USA), GraphPad Prism 9.0 (GraphPad, San Diego, CA, USA), and IBM SPSS 20.0 (IBM, Armonk, NY, USA). The significant differences (*p* < 0.05) shown in this study were calculated using statistical method of analysis of variance (ANOVA) tests. The data are presented as the mean ± stand error.

## 5. Conclusions

In this study, *Mtr-miR319a*, a miRNA considered to participate in salt response and tolerance in *Medicago* species, as per our prior report, was overexpressed in *A. thaliana* and *M. truncatula* plants. Our results showed that overexpressing *Mtr-miR319a* induced leaf curling phenotypes and enhanced salt tolerance in both the *A. thaliana* and *M. truncatula* overexpression plants. The expression of *Mtr-miR319a* was determined to be maintained at a high level before and after salt stress, indicating the definite roles that *Mtr-miR319a* played in the process of leaf development and salt stress adaptation. Furthermore, the expressions of potential target genes, such as *MYB*s, *CUC*, *TCPs*, and *LOXs*, were detected, and among them *TCP4*, *TCP10*, *MYB1*, *CUC2*, *LOX9*, and *LOX13* were found to be regulated by the overexpression of *Mtr-miR319a* and were suggested to be associated with the phenotypes observed in the *M. truncatula* plants; *TCP4*, especially, might be involved in controlling leaf margin formation and salt tolerance. These findings contribute to the molecular theoretical basis of *miR319* in legume plants and further provide important knowledge on developmental and stress tolerance traits for crop genetic engineering.

## Data Availability

Not applicable.

## Supplementary Materials

The following supporting information can be downloaded at: https://www.mdpi.com/article/10.3390/ijms24010429/s1.
