# Supplementary material for: Overexpression of Mtr-miR319a Contributes to Leaf Curl and Salt Stress Adaptation in Arabidopsis thaliana and Medicago truncatula
Source: Int J Mol Sci. 2022 Dec 27;24(1):429. doi: 10.3390/ijms24010429 (PMC9820427; doi:10.3390/ijms24010429)
Supplement: Supplementary file 1 [file ijms-24-00429-s001.zip › ijms-2072251-supplementary.pdf]

Table S1. Primers used in this study.

Primers	Sequences (5'- 3')
GSP-TCP4	CCGTCTGCAACGCAAACCGTGAACCTG
RNA adaptor	CGACUGGAGCACGAGGACACUGACAUGGACUGAAGGAGUAGAAA
5' universal primer	CGACTGGAGCACGAGGACACTGA
Mtr-miR319a-F	AAGTTCATTTCATTTGGAGAGATTAAAATTCTGGGTTTGG
Mtr-miR319a-R	TTTAAAGAAAGATCAAAGCTCACATTGCAGACACTTTGGC
Mtr-miR319a-3p	GTGGTTGGACTGAAGGGAGCT
U6	CGCACAAATCGAGAAATGGTCC
Mt-TCP4-F	ATGGGAGAAACAATACACGACC
Mt-TCP4-R	ATGGCGAGAGTCGGAGGAAG
qMYB1-F	GTTGGAGCTCCCTTCACTCC
qMYB1-R	AGCACTGCTCATGCCAATCT
qMYB2-F	TAACAGCGGCAACAAAGTGC
qMYB2-R	AGATGTGCCCCAGCTACCTA
qCUC2-F	GGAGCACGTGTCCTGTTTCT
qCUC2-R	TGTTGAGACCACCACCACTG
qTCP3-F	TCGACTGGGCTTTGATAGGC
qTCP3-R	ACGACGCCGTTTCAGGTATT
qTCP4-F	GCTATCGACGAACTCGCTCA
qTCP4-R	TTTCCGGCGAAAAGCACTTG
qTCP10-F	ATAGGGGGACCCTTCAGTCC
qTCP10-R	CCATGGCTCTCATCCTCACC
qLOX9-F	ACGTGCGGTCACTTTACGAT
qLOX9-R	CCTCATCAGTCATCCACGCA
qLOX13-F	AAGCATGGTGGACAGAAGTT
qLOX13-R	TCTCAGCCCATGATGGTTCA
qACTIN2-F	CAAAAGATGGCAGATGCTGAGGAT
qACTIN2-R	CATGACACCGGTATGACGAGGTCG
